# Impact of NMDA receptor activation on DNA damage in PC12 neuron-like cell cultures in the presence of β-amyloid peptides

**DOI:** 10.1007/s11033-022-07856-6

**Published:** 2022-09-15

**Authors:** Benita Wiatrak, Przemysław Mieszała, Kazimierz Gąsiorowski

**Affiliations:** 1 Department of Basic Medical Sciences, Wroclaw Medical University, Borowska 211, 50-556 Wroclaw, Poland; 2 Department of Pharmacology, Faculty of Medicine, Wroclaw Medical University, Mikulicza-Radeckiego 2, 50-345 Wrocław, Poland

**Keywords:** Alzheimer’s disease, Long-term potentiation, NMDA receptor, β-amyloid

## Abstract

**Objective:**

This study aimed to investigate the effect of low nanomolar concentrations of Aβ1–40 and Aβ25–35 on DNA double-strand breaks following NMDA activation of cells.

**Materials and methods:**

After incubating the differentiated PC12 cells with Aβ_25−35_, Aβ_1−40_ or Aβ_1−42_ for 24 h, the culture was washed and stimulated for 15 min with NMDA. Then, tests were performed at four-time intervals from stimulation to assess the viability of the culture, the level of oxygen free radicals, and the γH2AX and pATM kinase. NMDAR1 expression was also evaluated by performing immunocytochemical staining.

**Results:**

It was found that amyloid peptides in nanomolar concentrations reduce double-stranded DNA breaks after NMDA neuron activation. A slight antioxidant effect was also demonstrated when measured 120 min after NMDA cell activation.

**Conclusion:**

The NMDA stimulation of PC12 cells led to a rapid increase in the number of double-stranded DNA breaks in the cells and is assumed to be the initial step in IEG activation and LTP induction. The effect of Aβ on the reduction of double-strand breaks after NMDA cell stimulation indicates that at concentrations similar to physiological amyloid peptides, it may reduce the mobilization of the neuronal response to stimuli, leading to inhibition of LTP induction and decreasing synaptic plasticity in the early stages of Alzheimer’s disease.

## Introduction

Amyloid deposition is still recognized as the most popular cause of Alzheimer’s disease. Amyloid-β is not a single molecule, but we distinguish between different isoforms that vary in length. They can be 39 to 42 amino acids long [1]. Other forms of amyloid have various activities, and the way they work is also concentration-dependent. Amyloids can be both antioxidants and show neuroprotective and harmful properties. The beneficial properties are present at doses ranging from pico to nanomolar, that is, at physiological concentrations and soluble forms. Amyloid toxicity occurs at higher doses, affects the insoluble forms, and involves various mechanisms. These amyloids can induce oxidative stress, mitochondrial dysfunction, changes in membrane permeability, and excitotoxicity [1]. Aβ_1-40_ is the most abundant isoform in the brain (80–90%), while Aβ_1-42_ is much less widespread but increases significantly in Alzheimer’s disease. It is more hydrophobic and fibrillogenic, which causes an increased tendency to aggregate and neurotoxicity [2].

However, the dose is said to make the poison – studies suggest that Aβ_1-42_ may be neuroprotective at very low concentrations. This is because it is a very effective antioxidant due to its high affinity for redox-active copper and is important in the pathogenesis of Alzheimer’s disease. At average concentrations, Aβ_1-40_ is much less toxic than Aβ_1-42_. Aβ_1-40_ prevents cell damage caused by Aβ_1-42_ and the formation of fibrils and is also an antioxidant [3–6].

Other types of amyloid-β, such as Aβ_25-35_ and Aβ_31-35_, showed no neuroprotective properties and even turned out to be potent neurotoxins, even in a disaggregated form. These short-chain amyloids enter the cell contributing to the destruction of mitochondria and the enhancement of pro-apoptotic signals [7]. Aβ_25-35_ increases the ejection and inhibits the uptake of glutamate and aspartate, related to this amyloid’s induction of free radicals. It also causes an increase in intracellular calcium concentration as well as excitotoxicity and, consequently, cell death. After some time of exposure to this amyloid, the surviving cells develop resistance and their glutamate uptake increases [8]. The above information shows that not only the amount of Aβ is important in the pathogenesis of Alzheimer’s disease, but also its type [7].

An essential process of synaptic plasticity is long-term potentiation (LTP), which increases the strength of synaptic connections between neurons. This process is the main cellular mechanism responsible for learning and memory. Many studies have shown that memory impairment in the early stages of Alzheimer’s disease can be caused by LTP inhibition by excess β-amyloid (Aβ) [9].

AMPA and NMDA receptors play a crucial role in the induction of synaptic plasticity, but their cooperation is essential, and they cannot induce LTP alone. AMPA depolarisation unblocks NMDA receptors, allowing the influx of Ca^2+^ ions into the interior of cells, which are an essential mediator of LTP expression [10–12]. Calcium activates Ca^2+^/calmodulin-dependent protein kinase II (CaMKII), which phosphorylates AMPA receptors by increasing their permeability to ions and intensifying ions’ insertion into the cell membrane. This process facilitates further depolarization and activation of the NMDA receptor and the translation of dendritic mRNA and cytoskeleton remodeling [13, 14]. Calcium also activates the Raf/MEK/ERK signaling pathway, which can induce the expression of immediate early genes (IEGs) such as Fos, FosB, c-Jun, Npas4, Egr1, Nr4a1, Nr4a3. These genes are involved in the neuronal growth, development, and maturation of synapses and in maintaining a balance between excitatory and inhibitory synapses [15, 16]. Raf/MEK/ERK signaling pathway, as well as PKA and PKC kinases, induce phosphorylation of CREB protein – a transcription factor that enhances the expression of many IEGs (including c-Fos, c-Jun, cMaf, FosB), many neuropeptides and PPAR transcriptional factors that are involved in the formation of LTP [17, 18]. They also increase the expression of the Arc gene, whose mRNA is transported to dendrites, where its translation occurs. The Arc gene’s mRNA transport complex is docked near the presynaptic membrane using F-actin filaments, which are necessary for LTP stabilization (extension of dendritic spines with an accumulation of F-actin). The Arc protein enhances the process of F-actin accumulation, so there is a positive feedback mechanism [19, 20].

Intensification of IEG expression is one of the key processes in the early stages of LTP. However, their transcription by RNA polymerase II is blocked due to the topological constraints associated with DNA supercoiling. Studies have shown that cognitive activity related to coding new information causes quickly repairable DNA double-strand breaks (DSBs). Most of them arise in IEG promoters and are mediated by IIβ topoisomerase [7, 16]. Through such precise enzymatic incisions, the topological restrictions of IEG promoters are relaxed, and transcription occurs. The resulting proteins are involved in activating secondary response genes (SRGs) and further LTP stabilization.

LTP processes are disturbed in Alzheimer’s disease. The β-amyloid at micromolar concentrations (especially in oligomeric and fibrillar forms) leads to ERK kinase hyperphosphorylation, CBP protein accumulation, inhibition of CREB phosphorylation, and inhibition of CREB-dependent genes, and can affect most of the Raf/MEK/ERK-mediated metabolic pathways [17, 21]. Aβ directly blocks synaptic NMDA receptors and activates metabotropic glutamate receptors, resulting in increased internalization of AMPA receptors [22–24]. In addition, arc protein stimulates the γ-secretase complex by binding to presenilin 1, enhancing the formation of Aβ, which can increase the expression of the Arc gene. These facts suggest the presence of positive feedback, resulting in an intensification of long-term depression (LTD, opposing process to LTP) due to excess Arc protein [20, 25]. Aβ also inhibits DSB repair mechanisms, causing damage accumulation and intensification of degenerative processes [7].

Damage to DNA in a cell is common. It is estimated that a million single-stranded DNA damage occurs every day, statistically in every cell. Even if the cell is not exposed to genotoxic compounds, chemical or ionizing radiation, the very structure of DNA makes it not completely stable. This is the case, for example, with the linkage between deoxyribose and adenine or guanine, which are very susceptible to hydrolysis, which may favor the formation of a mutation. Additionally, the products of cellular metabolism, including free radicals and reactive oxygen species like hydrogen peroxide, can also damage the genetic material [26]. If this damage is not repaired, it can cause cell dysfunction, death, or cancer initiation. The cell has developed many mechanisms to repair DNA damage. Which one will be used depends on many factors, including the type of damage.

One way to assess the amount of DSBs in cell culture is to evaluate the activation/phosphorylation of ataxia-telangiectasia mutated kinase (ATM) and histone H2AX. ATM is one of the first kinases activated after the appearance of DSBs. It is also one of the main factors responsible for rapid histone H2AX phosphorylation at Ser139 [27]. DNA is kept in chromatin in nucleosomes, made of double-stranded DNA with a negative charge and proteins – histones with a positive electric charge. Phosphorylated histone H2AX (γH2AX) spreads along the chromosome and accumulates at sites of double-strand DNA damage. Although γH2AX is not necessary to carry out DNA repairs, it significantly accelerates them, stabilizing and maintaining the structures of DNA repair foci [27].

Our research examined the effect of low nanomolar Aβ concentrations on neuron activation processes and DSB formation in neuron-like PC12 cells. The study allowed us to investigate the potential impact of Aβ on LTP processes in the early stages of Alzheimer’s disease, which corresponds to the tested range of Aβ concentrations.

## Materials and methods

### Tested compounds

The *N*-Methyl-D-aspartic acid (NMDA) and β-amyloid (Aβ) 1–40, 1–42 and 25–35 were purchased from Sigma-Aldrich. The NMDA was dissolved in distilled water to the stock concentration of 20 mM. Aβ_1-40_, Aβ_1-42_ and Aβ_25-35_ were also dissolved in distilled water to the stock concentration of 1 mM. All stock solutions were stored at -20 °C for up to 6 months.

NMDA was used in assays in the concentration range of 1-1000 µM and β-amyloid (Aβ_1-40_, Aβ_1-42_ and Aβ_25-35_) in concentrations of 10-1000 nM. Aβ was dissolved to the final concentrations at room temperature for 15 min, and prepared concentrations were added to the cell culture 30 min after pulling out the freezer.

### Culture medium

RPMI-1640 medium was supplemented with 10% DHS (donor horse serum), 5% FBS (fetal bovine serum), 2 mM L-glutamine, 1.25 µg/ml amphotericin B and 100 µg/ml gentamicin. The prepared culture medium was stored at 4–8 °C for up to 1 month. To differentiate PC12 cells, the medium was additionally supplemented with 100 ng/ml of nerve growth factor (NGF) and with a reduced amount of serum to 5% of DHS alone.

### Culture plates

Collagen type I was obtained from Sigma-Aldrich and dissolved in 0.1 M acetic acid to get a 0.1% collagen solution, stored at -20 °C.

The plates were covered with collagen type I to induce cells’ adherence to the wells’ surface. Collagen solution was diluted with water to a final concentration of 0.01% and applied to wells. The plates were incubated overnight at 4–8 °C. After this, wells were washed with PBS three times, and leaves were stored at 4–8 °C for one month. Before use, the plates were irradiated with UV for 30 min.

### Cells and conditions

The study used the PC12 cell line (rat pheochromocytoma) obtained from ATCC. This cell line is widely used to study the mechanism of Alzheimer’s disease, particularly the mechanisms of neurotoxicity. After differentiation with NGF, these cells have properties similar to neurons, e.g., the ability to produce and store catecholamine, produce a secretory form of APP, and respond to treatment with Aβ. The PC12 cells were incubated under 5% CO_2_ and 95% humidity at 37 °C. Cells were passaged twice a week by centrifugation at 1000 x g for 5 min. Then the supernatant was removed, the fresh medium added, and the cell clumps disrupted by double passing through the 0.7 mm needle.

The cell suspension was prepared for LDH and DCF-DA assays at the density of 20 × 10^3^ cells/well (96-well plate) and for DNA damage assessment at the density of 100 × 10^3^ cells/well (12-well plate). After seeding, the cells were incubated overnight for regeneration and adhesion of cells to the well surface.

### Assays

To study the effect of two β-amyloid fragments (Aβ_25-35_, Aβ_1-40_ and Aβ_1-42_) on the LTP process induced by activation of NMDA receptors, cell viability (LDH assay), and the level of reactive oxygen species (DCF-DA assay), and DNA damages (ATM and H2AX activation) were examined.

First, the effects of amyloid and NMDA used separately were investigated. Assays were carried out after 24-hour incubation of PC12 cells with Aβ_25-35_, Aβ_1-40_ or Aβ_1-42_ at selected concentrations in the range of 10-1000 nM. NMDA was used at concentrations of 1-1000 µM with an incubation time of 15 min. Then the supernatant was removed, and a fresh medium was added for 24 h. After this time, tests assessing the impact of NMDA were carried out. The control group (negative) was cell culture without compounds.

After performing the above-described tests assessing the effects of NMDA and β-amyloid, a concentration range of 10–100 nM was selected for the main stage of the study for both Aβ_1-40_ and Aβ_25-35_, and a concentration of 75 µM for NMDA. The cell culture was treated with the selected attention of each amyloid for 24 h. The supernatant was then removed, the cultures washed, and the NMDA solution (75 µM) added for 15 min. Assays were performed immediately after NMDA removal (and washing with PBS) or after additional incubation in a culture medium for 15, 45, or 105 min, respectively. In this way, measurements were obtained after 15, 30, 60, and 120 min from the moment of cell activation. Negative control in this part of the study was the cell culture also incubated with NMDA but pre-incubated without β-amyloid.

### Cells viability

The LDH assay was performed using Pierce LDH Cytotoxicity Assay Kit (Thermo Scientific, USA). After incubation with compounds according to procedures described earlier, the supernatant was collected and transferred to another plate. The reaction mixture was added to plates with the collected supernatant, and the plates were left in the dark for 30 min to allow the reaction to proceed. A stop solution was then added to terminate the response, and the absorbance measurement was performed at 490 and 680 nm in a microplate reader (Victor2, Perkin-Elmer).

### Reactive oxygen species (ROS) level

The reactive oxygen species (ROS) level was measured using the DCF-DA assay. After incubation with compounds, the culture medium was removed, and cells were washed in PBS. Then 1 × 10^-6^ M DCF-DA solution in MEM (without serum) was added for one h at 37 °C. Fluorescence was measured with excitation at 485 nm and emission at 535 nm using a microplate reader (Victor2, Perkin-Elmer). In addition to the negative control, positive control was also used by adding 100 µM H_2_O_2_ for the last hour of incubation.

### DNA damages

DNA damages were measured by evaluating ATM and H2AX activation/phosphorylation using the Muse Multi-Color DNA Damage Kit (Merck Millipore, USA). First, cells were detached with a 0.01% TrypLE solution in PBS for 20 min. When β-amyloid fragments and NMDA were examined separately, the cells were immediately centrifuged at 1000 x g for 5 min. However, in the main stage of the study, after detaching cells from multiwell plates, NMDA [75 µM] was added to cells and incubated for 15 min. The experiment was then carried out in the same manner independently of the stage of the study. After centrifuging, a 50% fixation buffer in 1x Assay Buffer for 10 min on ice was added to the cells. Next, cells were centrifuged under the same conditions, the supernatant removed, and 100% cold Permeabilisation Buffer added for 10 min on ice. Next, cells were centrifuged at 300 x g for 5 min and resuspended in the antibody mixture for 30 min in the dark at RT. Finally, after another centrifugation, the cells were resuspended in Assay Buffer, and the amount of phosphorylated histone H2AX (γH2AX) and ATM (pATM) were analyzed using Muse Cell Analyzer.

### Expression of the N-Methyl-D-Aspartate (NMDA) receptor

Cell cultures were fixed with 4% paraformaldehyde for 30 min at room temperature after 15, 30 and 60 min of activation with 75 µM NMDA. The cells were then washed 3 times with PBS.

Permeabilization was performed using a solution of 0.1% Triton X-100 in PBS for 10 min at RT. Subsequently, nonspecific bindings were blocked by 30 min incubation with a PBS solution supplemented with 1% bovine serum albumin (BSA) and 10% normal goat serum (NGS). The primary antibody (Cat # GTX133097) was then administered overnight at a concentration of 1: 500 at room temperature. The next day, the culture was washed 3 times with PBS for 5 min, and the secondary antibody was administered at a dilution of 1: 250. Finally, pictures were taken using the EVOS FL microscope.

### Statistical analysis

The results are presented as E/E_0_ ratios, where E is the result of a tested compound, and E_0_ is the negative control used in a given study stage. The Shapiro-Wilk test for obtained results was carried out to assess normality distribution and Levene’s test to determine the homogeneity of variance. These tests showed that parametric statistical tests could be used to analyze the results. One-way ANOVA and Tukey’s post-hoc test were carried out using Statistica 13.3 (StatSoft, USA).

## Results

### β-amyloid and NMDA were used separately

#### Cells viability

**Fig. 1:**
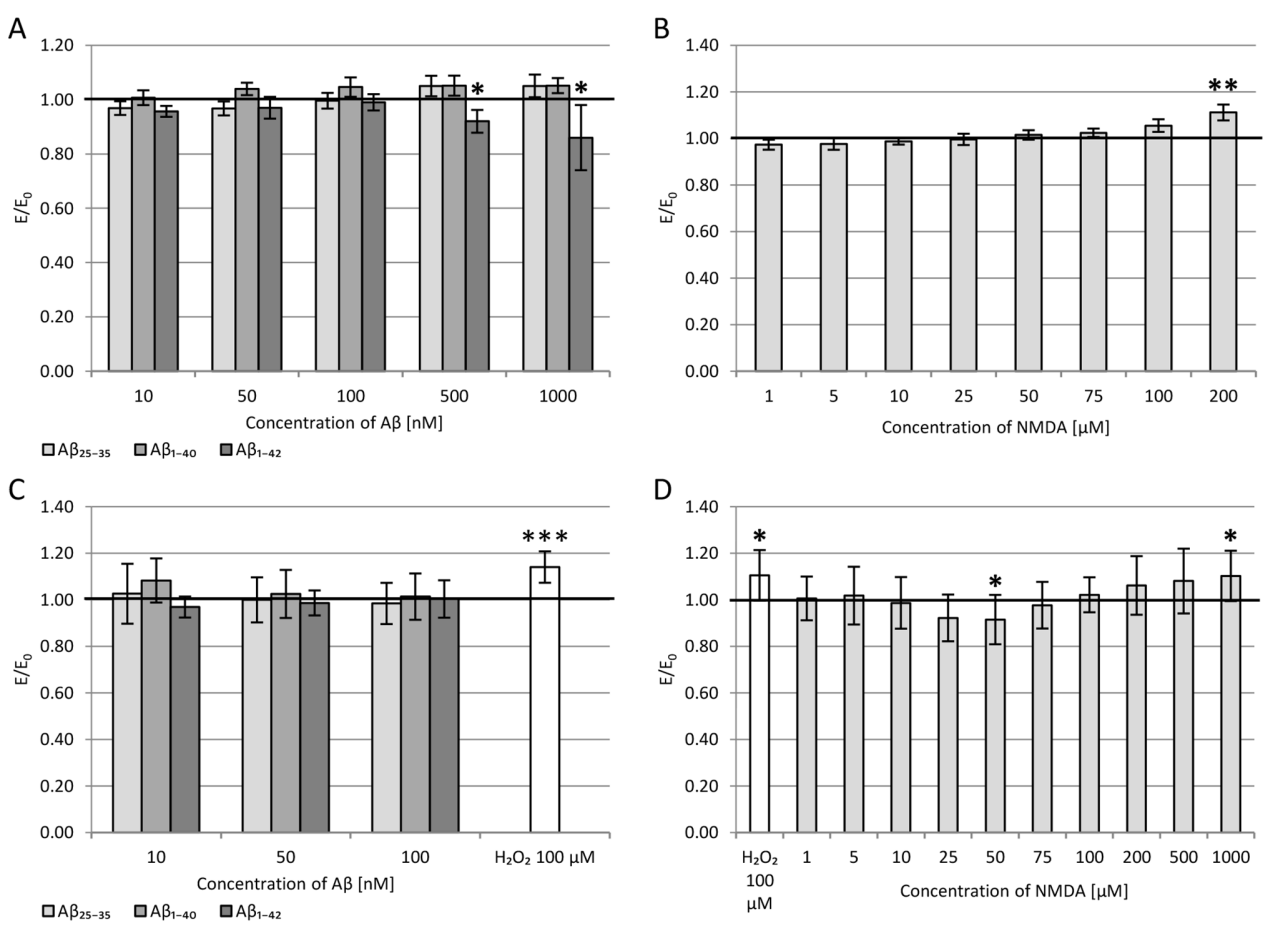
Effect of β-amyloid (A and C) and NMDA (B and D) on the viability of PC12 cells measured by LDH release (A and B) and the intracellular ROS level (C and D). Results were compared to the control without β-amyloid and NMDA, and are presented as mean ± SEM (* p < 0.05; ** p < 0.01; *** p < 0.001). The positive control in the DCF-DA assay (C and D) was culture incubated with H_2_O_2_ [100 µM, 30 min] without β-amyloid and NMDA

The viability of PC12 cells after 24-hour incubation with Aβ_25-35_ or Aβ_1-40_ or Aβ_1-42_ in 10-1000 nM concentrations was assessed by LDH assay (Fig. 1 A), which showed no statistically significant effect of βamyloid on cell viability compared to control for fragments 25–35 and 1–40. However, significant cytotoxicity was statistically observed at 500 and 1000 nM for Aβ_1-42_.

The evaluation of PC12 cells viability after 15 min incubation with NMDA at concentrations of 1-200 µM and then 24-hour incubation with culture medium was also determined by LDH assay (Fig. 1B). As the concentration increased, an increase in LDH release was observed, i.e., a decrease in viability – statistically significant for a concentration of 200 µM.

#### Reactive oxygen species (ROS) level

All tested Aβ fragments’ effects on reactive oxygen species (ROS) levels were determined using the DCF-DA assay (Fig. 1 C). In the range of Aβ concentrations tested, there was no significant increase in ROS level. However, incubation of PC12 cells with 100 µM H_2_O_2_ for 30 min (positive control) resulted in a substantial increase in oxygen free radicals (approximately 14%).

The NMDA in PC12 cells did not affect ROS levels at low (1–10 µM) and high (100–200 µM) concentrations (Fig. 1D). In contrast, in medium concentrations (25–75 µM), a pronounced decrease in the amount of oxygen free radicals was observed. At 50 µM of NMDA, the reduction was about 10% compared to the control (without NMDA) and was statistically significant. Incubation with 100 µM H_2_O_2_ for 30 min (positive control) increased ROS by about 11%. At the highest tested concentration of 1000 µM NMDA, an increase in the ROS level was observed, similar to that in the case of the H_2_O_2_ incubation.

#### DNA damages

The effect of 24-hour incubation with β-amyloid (Aβ_1-40_ or Aβ_25-35_ or Aβ_1-42_; 10–100 nM) or NMDA (501,000 µM) on the phosphorylation/activation of both H2AX and ATM was evaluated.

**Fig. 2:**
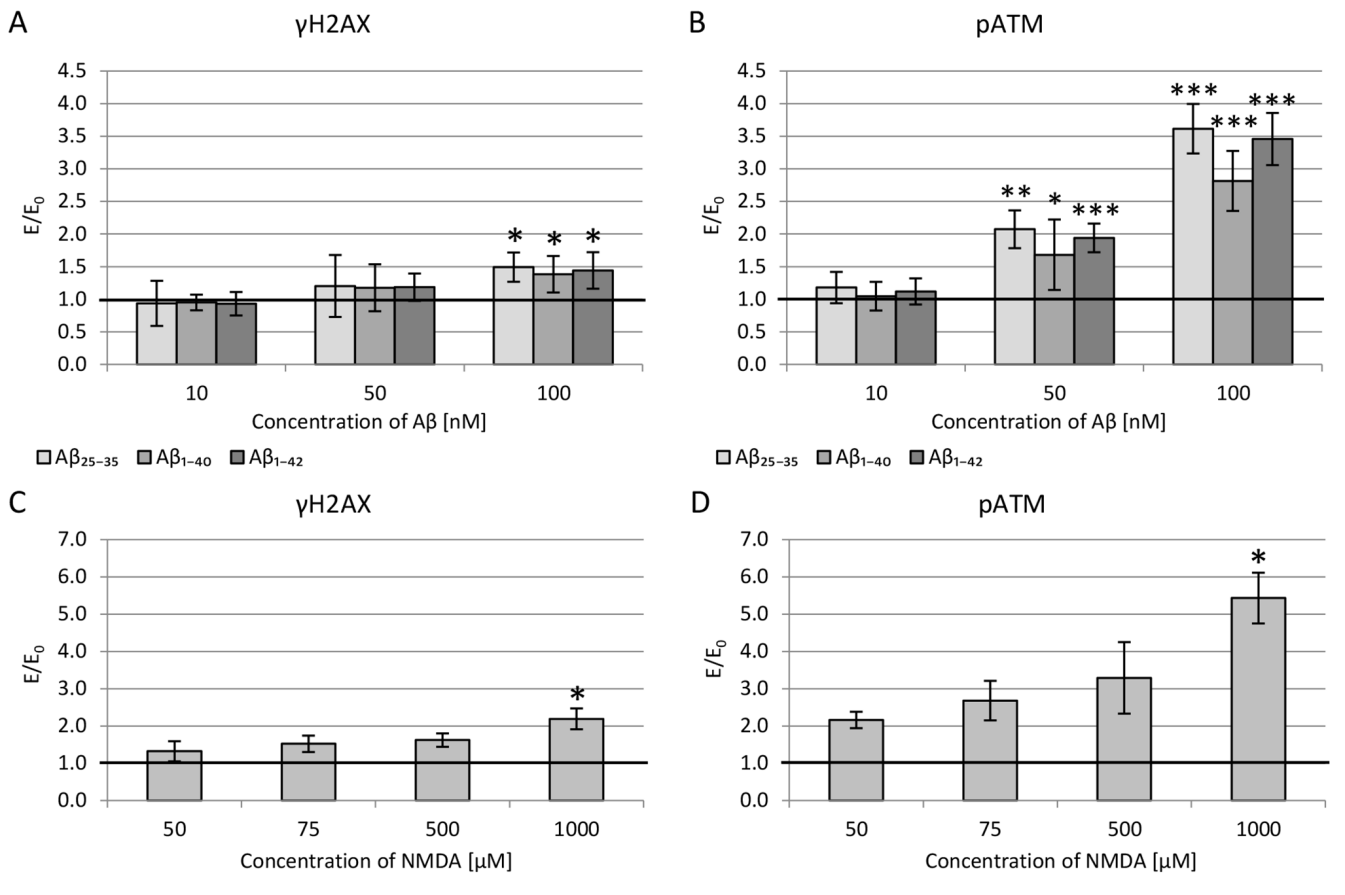
DNA damage assessment: histone H2AX phosphorylation (γH2AX) (A and C) and ATM kinase activation (pATM) (B and D) in PC12 cells incubated with β-amyloid (A and B) or NMDA (C and D). Results were compared to the control without β-amyloid and NMDA, and are presented as mean ± SEM (* p < 0.05; ** p < 0.01; *** p < 0.001)

In all cases studied, the effect was concentration-dependent – the level of H2AX and ATM activation increased with compound concentration (Fig. 2 A and B). A statistically significant increase in DSBs (phosphorylated H2AX) was observed at 100 nM of all β-amyloid fragments. In addition, a substantial level of ATM activation was observed at 50 and 100 nM concentrations.

In the case of NMDA, significant activation of H2AX and ATM occurred only at the highest concentration tested – 1000 µM (Fig. 2 C and D).

### Effect of β-amyloid on cell activation

After performing tests assessing the effects of NMDA and β-amyloid used separately, in the second stage of the study, the impact of β-amyloid (Aβ_1-40_, Aβ_25-35_ or Aβ_1-42_) at 10–100 nM concentrations on PC12 cells activation by NMDA (75 µM; 15 min) was examined. All assays were performed at four different periods after cell activation by NMDA (15, 30, 60, and 120 min). Concentrations of 10.50 and 100 nM were selected for further studies to be close to physiological concentrations so as to check whether amyloid inhibits the antioxidant and regenerative effects of DNA strand damage after exceeding the physiological concentration. The concentration of 75 µM NMDA was selected, which did not significantly increase the release of lactate dehydrogenase in the LDH assay, while ensuring the strongest cell activation.

#### Cells viability

**Fig. 3:**
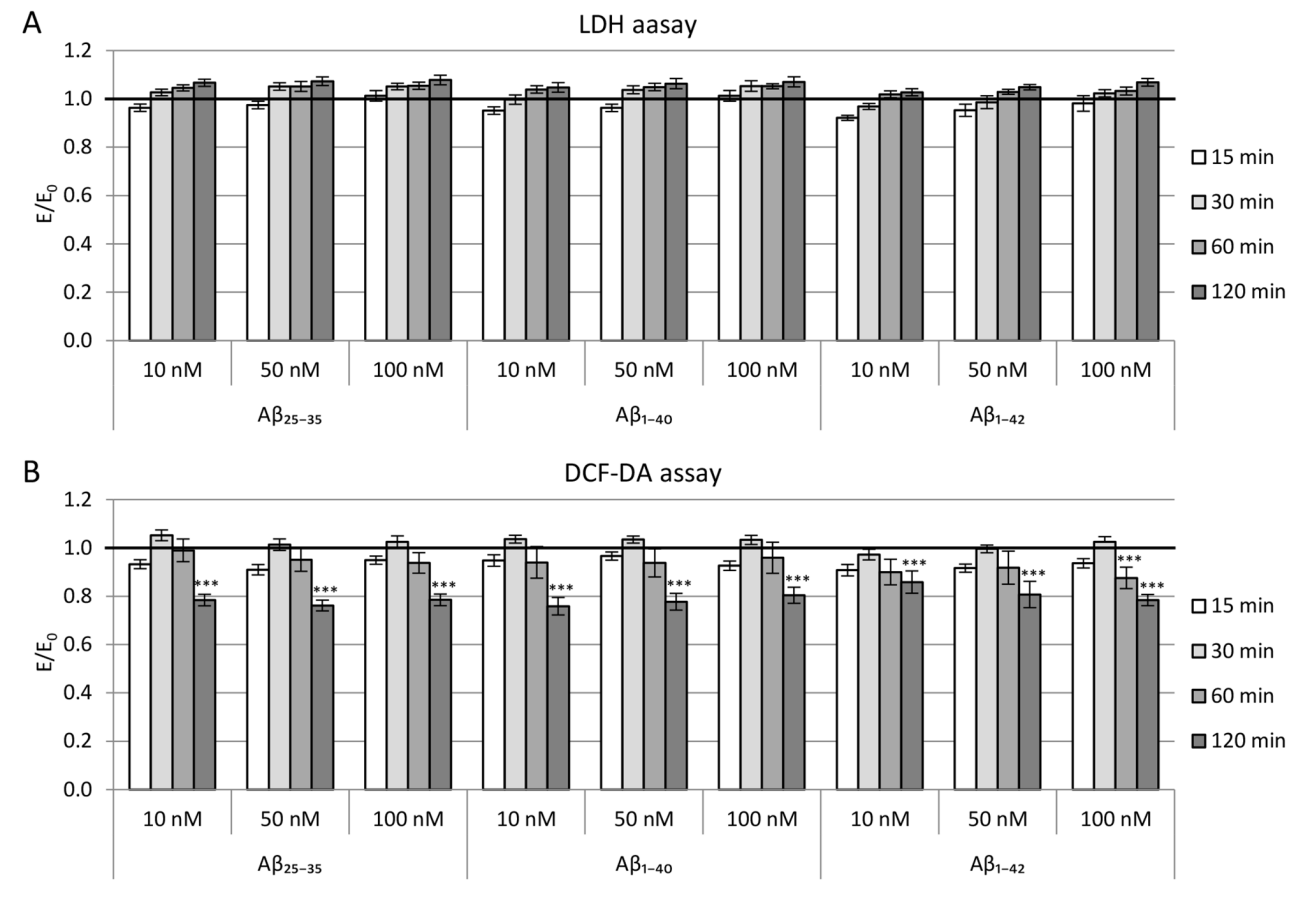
LDH release (A) and levels of intracellular ROS measured by DCF-DA assay (B) in PC12 cells incubated for 24 h with Aβ1–40, Aβ25–35 or Aβ1–42 and subsequently activated by NMDA (75µM; 15 min). Measurements were performed 15, 30, 60, and 120 min after cell activation. Results were compared to the control without β-amyloid but with cell activation by NMDA. Data are presented as mean ± SEM (*** p < 0.001)

The results of the LDH assay (Fig. 3 A) show no statistically significant effect of the tested βamyloid peptides on the viability of NMDA-activated PC12 cells compared to the negative control.

#### Reactive oxygen species (ROS) level

Levels of intracellular free radical content measured by DCF-DA assay are shown in Fig. 3B. The results indicate that incubation of the culture with β-amyloid reduced the ROS level in PC12 cells. For all Aβ_1-40_, Aβ_25-35_ and Aβ_1-42_, the level of free radicals 120 min after activation by NMDA was statistically significantly lower (by 20–28%) than in the control culture. The differences in ROS levels observed in previous measurements were not significant. Still, attention should be paid to a slight increase in free radicals level, which occurred 30 min after cell activation.

#### DNA damages

**Fig. 4:**
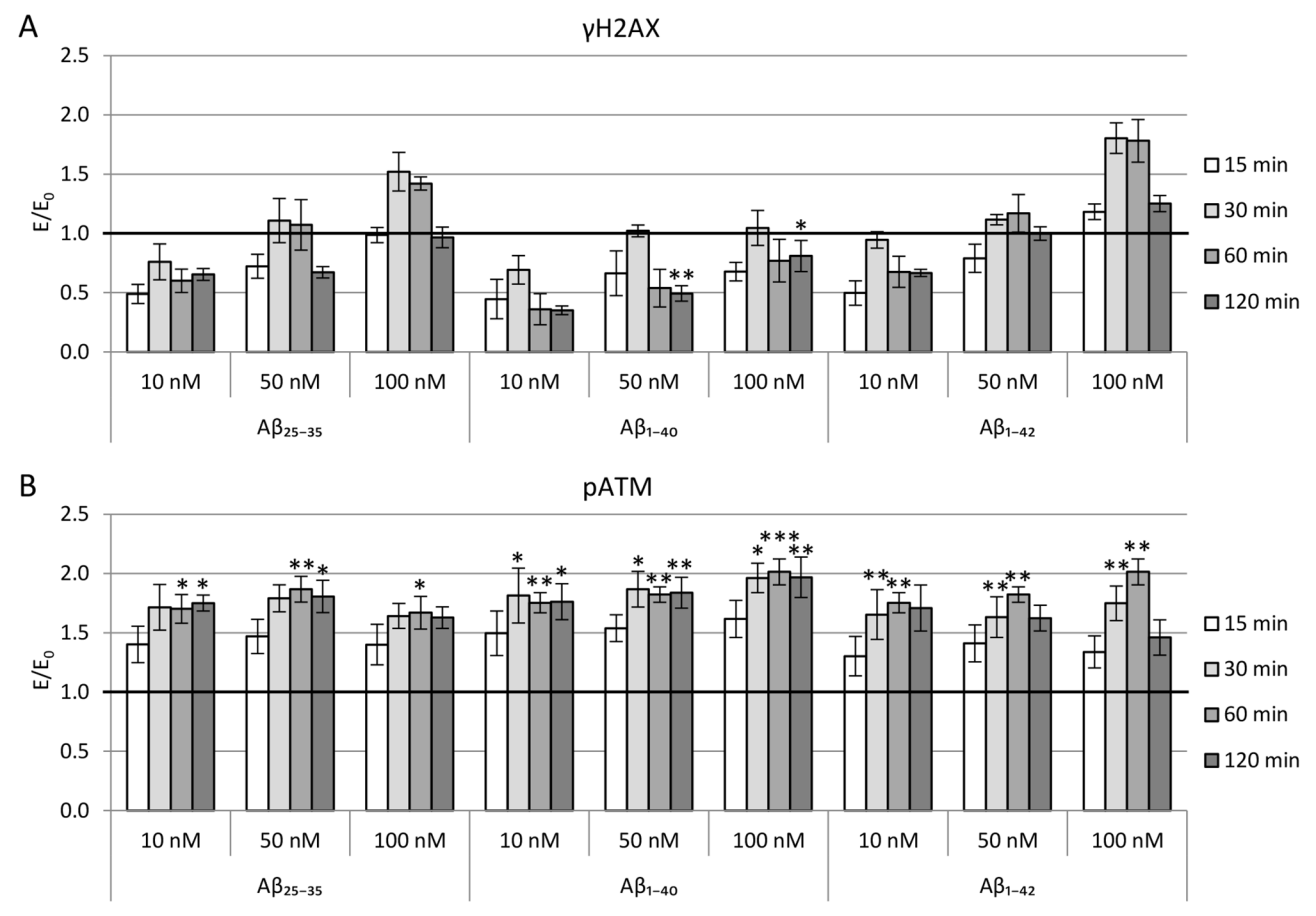
Evaluation of activation/phosphorylation of H2AX (A) and ATM (B) histone in PC12 cells incubated for 24 h with Aβ1–40, Aβ25–35 or Aβ1–42 and subsequently activated by NMDA (75µM; 15 min). Measurements were performed 15, 30, 60, and 120 min after cell activation. Results were compared to the control without β-amyloid but with cell activation by NMDA. Data are presented as mean ± SEM (* p < 0.05; ** p < 0.01, *** p < 0.001)

The evaluation results of ATM and H2AX activation/phosphorylation in NMDA-activated cells are shown in Fig. 4.

Preincubation with Aβ increased the phosphorylated ATM (pATM) level in culture, regardless of the type of β-amyloid, concentration, and measurement time (Fig. 4B). The increase of pATM was more pronounced for Aβ_1-40_ compared to Aβ_25-35_ or Aβ_1-42_. In measures 60 and 120 min after activation, differences were statistically significant – ATM phosphorylation levels increased 1.6x (Aβ_25-35_), 2x (Aβ_1-40_) and 1.8-2x (Aβ_1-42_). In the case of Aβ_1-40_, results obtained 30 min after NMDA administration is also statistically significant and are 81–96% higher than the control without Aβ.

The phosphorylation level of histone H2AX (γH2AX) strongly depended on the type of β-amyloid and its concentration (Fig. 4 A). The γH2AX level was higher in cultures with Aβ_25-35_ and Aβ_1-42_, regardless of attention and time of measurement. For all β-amyloid fragments, the level of H2AX activation increased with Aβ concentration. The lowest phosphorylation occurred at a concentration of 10 nM, at which the γH2AX level decreased for Aβ_2535_ by 30–40% and Aβ_1-42_ by 35% compared to the control, and for Aβ_140_ by as much as 40–65%. In cultures incubated with Aβ_25-35_ and Aβ_1-42_ at a concentration of 100 nM when measured 30 and 60 min after activation, a noticeable increase in H2AX activation (1.5x or 1.8x compared to the control, respectively).

#### Evaluation of NMDAR1 expression

**Fig. 5:**
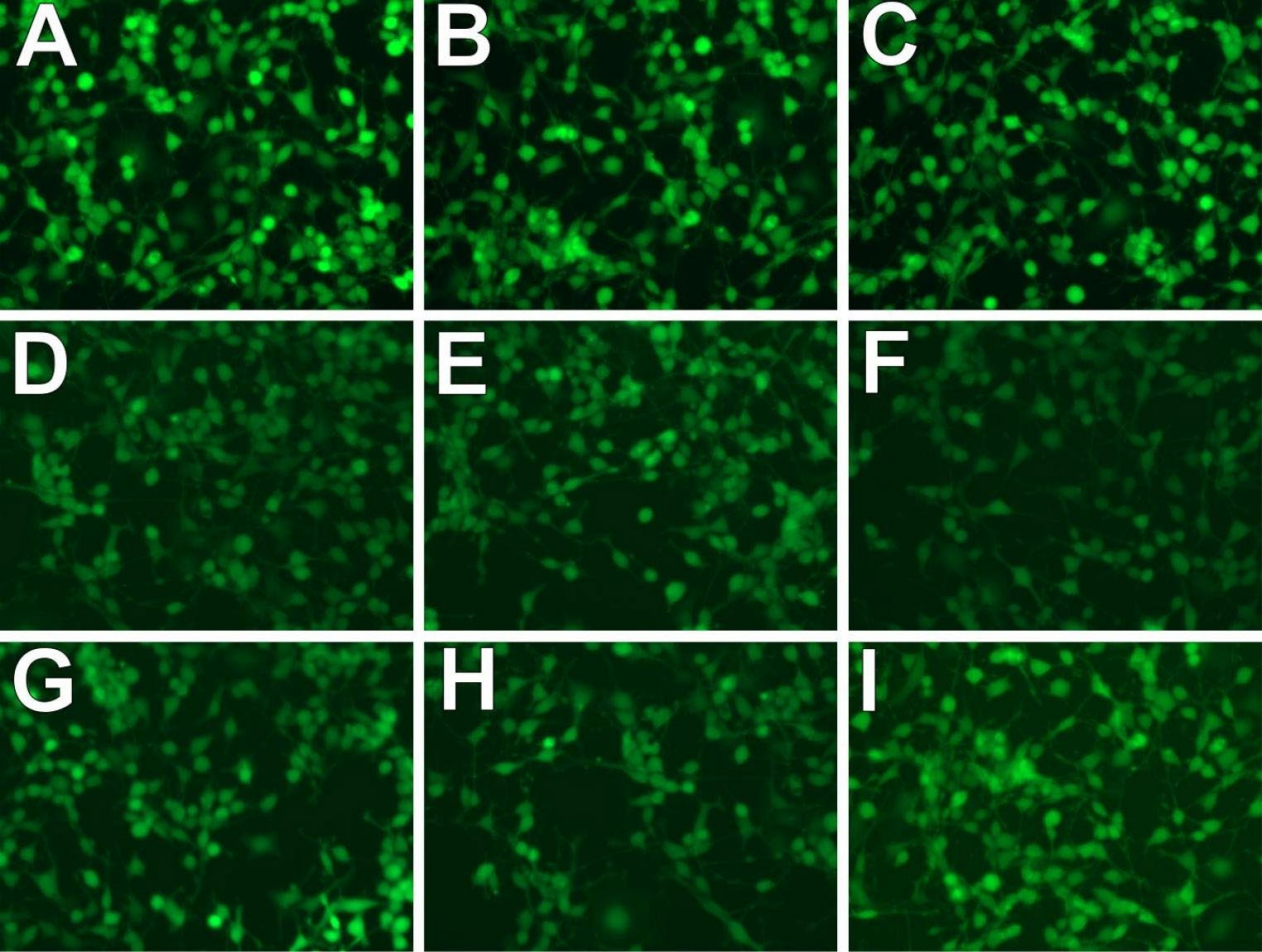
Changes in the level of expression of NMDAR1 in PC12 cells depend on the length of time since cell activation by NMDA [75 µM, 15 min]; A, D,G) Aβ_25−35_; B, E, H) Aβ_1−40_; C,F,I) Aβ_1−42_; A,B,C) after 15 min activation with 75 µM NMDA; DE,F) after 30 min activation with 75 µM NMDA; G,H,I) after 60 min activation with 75 µM NMDA

Immunocytochemical staining was performed to evaluate the expression level of the NMDAR1 receptor in cell cultures after 15, 30 and 60 min of incubation with 75 µM NMDA. As can be seen in Fig. 5, the intensity of NMDAR1 expression is significantly lower after 30 min incubation, irrespective of the selected amyloid fragment for pre-incubation. At the same time, one hour after the activation of NMDA culture, a slight increase in the expression of the receptor can be noticed (the highest in the case of an earlier pre-incubation with Aβ_1−40_).

## Discussion

Synaptic plasticity is defined as a fixed change in the efficiency of synaptic transmission. The cellular mechanisms of synapse plasticity include two main mutually opposite processes: long-term potentiation (LTP) and long-term depression (LTD). When learning and remembering, LTP is a process that plays an essential role in consolidating memory and adapting to changing conditions. The activation of many genes and neuronal metabolic pathways is required for LTP formation. An important role in activating these genes, especially in the early phase of LTP, is to play immediate early genes (IEGs). Their expression occurs quickly, immediately after the stimulus. Due to the packing/condensation of DNA and chromatin, breaking the topological barrier in regions of these genes is necessary. This process is possible through double-stranded DNA strand breaks (DSBs) locally relaxing chromatin structure [16]. Activation of neurons with physiological stimuli stimulates glutamatergic neuron networks. Almost immediately after glutamate binds to postsynaptic neuron receptors, topoisomerase IIβ is activated, cleaving the postsynaptic neuron’s DNA. This process causes double breaks in the promoter regions of various genes, especially often in the promoters of IEGs. Topoisomerase IIβ recruits DNA repair proteins to DSB sites and simulates RNA polymerase II and transcription factors [16], which leads to the activation of gene transcription below the site of DSB. The generation and repair of DSBs must be fast and effective (complete) because unrepaired breaks threaten genome stability and lead to mutation and/or cell death [16].

This study assessed the effect of Aβ_1-40_, Aβ_25-35_, and Aβ_1-42_ on the formation of physiological DSBs induced by the activation of cells. First, PC12 cell cultures were incubated with low (close to physiological) concentrations of β-amyloid peptides. The neuron-like cells were then briefly activated by incubation with the NMDA receptor agonist – *N*-Methyl-D-aspartic acid (NMDA).

The essential stage of the study was preceded by a preliminary assessment of the viability of the cells treated with the β-amyloid or NMDA separately to select non-toxic concentrations for PC12 cells. The results showed the non-toxicity of all Aβ fragments over the entire concentration range tested and NMDA at concentrations up to 100 µM. Furthermore, viability assessment in the main stage of the study (incubation with β-amyloid, followed by NMDA-induced cell activation) also indicated no cytotoxic effect of both compounds.

Literature data on the effect of NMDA on neuronal cells document different results (e.g., excitotoxicity in response to NMDA at a concentration from 20 µM to 8 mM [28–32]. The results of studies on the effect of β-amyloid peptides at micromolar concentrations on ROS levels in NMDA stimulated neurons (50–100 µM, 30 min) showed a significant increase in the level of free radicals in cells. It is known that the effect of compounds on cells depends on the concentration of these compounds, as well as on the type of cell culture and experimental system, the time and conditions of incubation with combinations, and in the case of amyloid peptides also on the degree of aggregation. The β-amyloid concentrations used in this study were many times lower than those commonly used in amyloid toxicity tests. These were mainly monomeric forms at the time of treatment in the cells. Aβ monomers were administered to the PC12 cell culture because they were administered immediately after preparation without prior pre-incubation of the amyloid solutions at 37 °C. Incubation of Aβ causes protein aggregation depending on the time from oligomers to fibrils [33–35]. The incubation time with NMDA was 15 min – shorter than usually used in research (30–60 min) [29, 30]. The goal was to provide PC12 cells with a powerful and sufficient impulse that mimics the effects of physiological stimuli, which are then subject to the LTP process and memorizing. The 50 µM NMDA concentration would seem attractive due to the cells’ significantly reduced free radical content. Still, sub-physiological levels of ROS are just as unfavorable for cells as their excess. The addition of antioxidants (reducing the level of ROS below physiological values) leads to dysregulation of ATM kinase and other enzymes involved in repairing DNA damage. Consequently, it increases the genomic instability of cells [36]. On the other hand, the theory of optimum redox potential suggests that minimal genomic damage arises at the physiological level of ROS quickly repaired and necessary to stabilize the LTP process [36, 37]. Therefore, the NMDA concentration selected in our research model (75 µM) seems optimal because it does not significantly affect the intracellular ROS level and does not reduce the viability of PC12 cells.

Numerous experimental studies confirm that β-amyloid induces the formation of DSBs in cells [7, 38] and inhibits DSB repair, leading to their accumulation and finally to cell apoptosis [16]. Most data indicate that the cytotoxic effect of Aβ occurs through the induction of oxidative stress in cells. This causes oxidative modifications of RNA, DNA, proteins, and lipids, considered the main pathomechanism of Alzheimer’s disease [29, 30, 39]. However, it is known that Aβ works not only by inducing oxidative stress. Recent studies show that soluble forms of Aβ_1-40_, Aβ_1-42_, and Aβ_25-35_ can interact directly with DNA, as proven by surface plasmon resonance [40, 41]. Aβ forms soluble but is unable to aggregate unable to bind DNA, suggesting a correlation between Aβ aggregation and their ability to bind DNA. Aggregated forms bind to DNA regardless of the degree of collection [40, 41]. These facts suggest that the ability to aggregate is an important factor but not the only one determining the binding of Aβ to DNA. Among the many amyloidogenic proteins in the body, the ability to bind to DNA is a characteristic of β-amyloid peptides [42].

To determine the effect of β-amyloid on DSBs induced by activation of PC12 cells with NMDA, in our study, the cells were incubated first with Aβ_1-40_, Aβ_25-35_ or Aβ_1-42_ (10, 50 or 100 nM) for 24 h and then with NMDA (75µM) for 15 min. The results showed a significant effect of the tested β-amyloid peptides on the level of activated ATM kinase (pATM) and DSBs measured by the amount of phosphorylated histone γH2AX. The story of pATM increased with the period from activation and reached a plateau after 30 min. The results confirm that Aβ increases cellular stimulation by activating metabolic pathways in which ATM kinase participates. However, the effect of the βamyloid on DSBs (amount of γH2AX) was the opposite – a significant reduction occurred, especially in cultures incubated with low, close to physiological concentrations of the peptide (10 nM).

As the concentration of amyloid β in the culture increased, the amount of DSB also increased, and the level of γH2AX increased most rapidly for Aβ_1-42_, then for Aβ_25-35_. The lowest increase amount of DSB lesions was observed for Aβ_1-40_. Differences between amyloid fragments may result from a much stronger tendency of Aβ_25-35_ and Aβ_1-42_ to aggregate into more toxic forms such as oligomers and fibrils [43]. DSB formation/repair kinetics shows an increase in double-strand breaks for 30 min after incubation with NMDA when it reaches a peak. In subsequent measurements, lower levels of γH2AX were noted. This trend occurred for all tested concentrations of both amyloid fragments and is probably the result of the rapid repair of DSBs caused by the physiological activation of neurons.

It is well known that at low (close to physiological) concentrations, Aβ inhibits DSB formation. The physiological role of amyloid peptides in LTP formation and synaptogenesis has been demonstrated in numerous studies [44, 45]. Earlier studies also showed a strong antioxidant effect by influencing the parameters of oxidative stress of Aβ at the physiological concentrations of all fragments (1–40, 25–35, 1–42) [5]. Additionally, the regenerative effect of Aβ 25–35 and 1–40 was observed on DNA strand damage caused by the earlier preincubation of PC12 cell culture with lipopolysaccharide [6]. It has also been disclosed that the lack of Aβ, obtained by its immunodepletion or secretase inhibition, causes cell death. This process can be inhibited by adding picomolar concentrations of Aβ to the culture [44].

In the experimental system used in our study, Aβ lowered the ROS level in all tested concentrations, with a statistically significant decrease after 120 min. Most literature data showing the peroxide effect of Aβ utilizes the micromolar concentrations of the peptide, which is additionally aggregated to oligomeric and fibrillar forms [46]. Our research used nanomolar concentrations of freshly prepared Aβ, i.e., mainly in monomeric forms. The literature also indicates the antioxidant effects of Aβ observed at pico- and nanomolar concentrations, and these effects disappear as the concentration of amyloid peptides increases [5]. The mechanism of β-amyloid antioxidant activity is probably related to the chelating properties of the peptide, which have an affinity, especially for copper ions, and iron ions, i.e., metal ions strongly catalyzing oxidative processes [46]. Therefore, the reduction of ROS in our study can be explained by the chelating properties of Aβ.

For all tested amyloid fragments (Aβ_1-40,_ Aβ_25-35_ and Aβ_1-42_), significant differences were observed between the effect on ROS and the level of double-strand breaks in DNA (γH2AX). However, all amyloids at each concentration tested had a similar impact on the ROS level. In contrast, the level of γH2AX depended on the attention and the type of βamyloid. These results indicate that the amount of DSBs in PC12 cells did not depend on the oxidative stress level. Therefore, the explanation of the mechanism of reducing DSBs formation in cultures incubated with Aβ and activated by NMDA may be internalization/endocytosis of NMDA receptors under the influence of Aβ and reduction of their number on the surface of the cell membrane [47]. Based on the analysis of the expression of NMDAR1 receptors, after the performed immunocytochemical staining, a decrease in the intensity of the expression of the receptors can be noticed – which may indicate a reduction in their number on the surface of the cell membrane. Probably, the incubation of PC12 cells with Aβ reduced NMDA binding to the NMDA receptor, which could result in the observed reduction of the NMDA effect on the formation of DSBs. Additionally, 30 min after NMDA administration, there was a relative increase in DSBs, indicating slower disclosure of the NMDA activation effect due to the reduced number of receptors.

After 60 and 120 min, there was a noticeable reduction in DSBs in all concentrations of all amyloid fragments. The decrease in the number of NMDA receptors does not explain this phenomenon because the increase in DSBs after 30 min indicates the activation of cells. Therefore, the results suggest that Aβ at the tested concentrations positively affects the mobilization of factors involved in DSB repair mechanisms. This effect was best seen at 10 nM Aβ concentration, which is closest to physiological. The fact that the most toxic fragment of β-amyloid – Aβ_25-35_ also lowers DSBs is surprising. However, this peptide is also expressed physiologically, indicating that it can perform biological functions at low concentrations [48]. As previously noted, the aggregation of Aβ increases with its attention, so it is possible that pico- and nanomolar concentrations of Aβ_25-35_ also have a positive effect at the molecular level, especially if they are in the form of monomers. At the same time, Aβ1–42 in concentrations of 10 and 50 nM influenced the activation of repair processes. At the highest concentration tested, fragments 1–42 showed the lowest regenerative activity of DSBs lesions. In our experimental system, at 10 nM Aβ, there was probably no significant fibrillisation of the peptide, and only higher concentrations resulted in greater aggregation, reflecting the increase in DSBs. Perhaps with an increasing concentration of Aβ, the amount of γH2AX would also increase, eventually accumulating DSBs and significant cytotoxicity.

In our study, in cultures incubated with Aβ and NMDA, the level of activated ATM kinase (pATM) increased significantly compared to the control without Aβ. However, a decrease in DSBs was noted. It is assumed that the activation of ATM kinase is associated mainly with phosphorylation/activation of proteins involved in repairing DSBs. However, the role of pATM in response to activation and cellular stress is much greater than participation in the repair of DNA damage. ATM kinase supports the cellular response to various genotoxic factors, often endogenous (including multiple types of DNA metabolism errors), but is also involved in cell signaling processes unrelated to DNA damage [49, 50]. In our experimental system, incubating PC12 cells with βamyloid and NMDA was a source of cellular stress, mobilizing ATM to participate in various pathways important for maintaining cell homeostasis. Therefore, the increase in pATM, significantly exceeding the level of histone H2AX phosphorylation, can be treated as the sum of activation of this phosphokinase in DSB recognition and repair processes and activation in the stress response pathways induced by the addition of Aβ and NMDA to the cell culture.
